# Diethylstilboestrol Exposure Does Not Reduce Testosterone Production in Human Fetal Testis Xenografts

**DOI:** 10.1371/journal.pone.0061726

**Published:** 2013-04-19

**Authors:** Rod T. Mitchell, Richard M. Sharpe, Richard A. Anderson, Chris McKinnell, Sheila Macpherson, Lee B. Smith, W. Hamish B. Wallace, Christopher J. H. Kelnar, Sander van den Driesche

**Affiliations:** 1 MRC Centre for Reproductive Health, Edinburgh University, Edinburgh, Scotland, United Kingdom; 2 Department of Child Life and Health, Edinburgh University, Edinburgh, Scotland, United Kingdom; Clermont Université, France

## Abstract

In rodents, *in utero* exposure to exogenous estrogens including diethylstilboestrol (DES) results in major suppression of steroidogenesis in fetal testes. Whether similar effects occur in the human fetal testis is equivocal. Based on the results of the rodent studies, we hypothesised that exposure of human fetal testes to DES would result in a reduction in testosterone production. We show, using a xenograft approach, that testosterone production is not reduced in human fetal testis following DES exposure. Human fetal testes (15–19 weeks’ gestation, n = 6) were xenografted into castrate male nude mice which were then treated for 35 days with vehicle or 100 µg/kg DES three times a week. For comparison, similar treatment was applied to pregnant rats from e13.5–e20.5 and effects on fetal testes evaluated at e21.5. Xenograft testosterone production was assessed by measuring host seminal vesicle (SV) weights as an indirect measure over the entire grafting period, and single measurement of serum testosterone at termination. Human fetal testis xenografts showed similar survival in DES and vehicle-exposed hosts. SV weight (44.3 v 26.6 mg, p = 0.01) was significantly increased in DES compared to vehicle-exposed hosts, respectively, indicating an overall increase in xenograft testosterone production over the grafting period, whilst serum testosterone at termination was unchanged. In contrast intra-testicular testosterone levels were reduced by 89%, in fetal rats exposed to DES. In rats, DES effects are mediated via Estrogen Receptor α (ESR1). We determined ESR1 protein and mRNA expression in human and rat fetal testis. ESR1 was expressed in rat, but not in human, fetal Leydig cells. We conclude that human fetal testis exposure to DES does not impair testosterone production as it does in rats, probably because ESR1 is not expressed in human fetal Leydig cells. This indicates that DES exposure is likely to pose minimal risk to masculinization of the human fetus.

## Introduction

Male reproductive disorders (cryptorchidism, hypospadias, testicular germ cell cancer, low sperm counts) are common and some have increased significantly in incidence in recent decades, raising concern about the role that lifestyle/environmental factors play in their etiology [1]. It has been proposed that these disorders are components of a ‘Testicular Dysgenesis Syndrome’ (TDS) with a common origin in fetal life [2]. Suppression of testosterone production resulting in abnormal testis development/function during fetal life has been implicated in the development of the TDS disorders [1], [3], [4], [5]. What remains unclear is what lifestyle/environmental factors could affect human fetal testis development/function and give rise to these disorders.

Twenty years ago the so-called ‘estrogen hypothesis’ was proposed which suggested that the increasing incidence of reproductive abnormalities in the human male may be related to increased estrogen exposure *in* [6]. This hypothesis was supported by a number of studies in pregnant rats exposed to the synthetic estrogen DES and by some epidemiological data in male offspring of mothers exposed *in utero* to DES [2]. Male offspring of rats exposed *in utero* to DES demonstrated gross suppression of testosterone [7], [8]. Studies in estrogen receptor-1 knockout (ERKO) mice have shown that the effects of DES are mostly mediated through classical estrogen signalling pathways involving ESR1 [9].

During the 1940’s–1970’s DES (∼60–70 µg/kg/d) was administered to pregnant women to prevent miscarriage and was reported to result in an increased incidence of reproductive tract abnormalities in both male and female offspring [10], [11]. However, other studies of the consequences of exogenous estrogen exposure in human pregnancy, including detailed meta-analyses, have largely failed to provide support for the estrogen hypothesis-TDS relationship in humans [12], [13], [14], [15]. Moreover, some studies have indicated that ESR1 is not expressed in human fetal Leydig cells [16], [17].

In view of the previous lack of a human relevant system in which to investigate the effects of *in utero* exposure to estrogens, there remains uncertainty about the risk that environmental estrogens pose to masculinization of the human male [2]. We hypothesised that exposure of human fetal testis to DES results in a reduction in testosterone production as it does in rats and mice. We have validated a xenograft system to determine the effects of exposure to environmental chemicals on the human fetal testis [18]. The system involves grafting human fetal testis tissue into immune-compromised castrate mice, where it grows and develops normally for prolonged periods (6+ weeks) [19]. Treatment with hCG during the grafting period to mimic normal *in utero* exposure, maintains Leydig cell steroidogenesis and thus stimulates growth of the host seminal vesicle, reflecting testosterone production from the xenografts over the duration of the grafting period [19]. The xenograft model more effectively recapitulates the normal situation in pregnancy, without the reservations about *in vitro* and indirect approaches and as a result has the potential to provide a more ‘physiological’ system in which to evaluate the effects of exposure to estrogens on human fetal testis testosterone production. This was the aim of the present studies.

## Methods

### Ethics Statement

For all studies, animals were treated humanely and with regard for alleviation of suffering. Studies were performed according to the Animal (Scientific Procedures) Act 1986 following specific approval by the UK Home Office. Studies were conducted under an approved Project Licence (PPL 60/3914) following review by the University of Edinburgh Animal Research Ethics Committee. In order to obtain human fetal testes, women undergoing elective termination gave written consent in accordance with UK national guidelines [20], and ethical approval for this study was obtained from the Lothian Research Ethics Committee (LREC - 08/S1101/1).

### Human Fetal Testes and the Xenografting Procedure

Second trimester (15–19 weeks, n = 6) human fetal testes were obtained following elective termination of pregnancy. No terminations were due to fetal abnormalities. A small portion of each testis was fixed as a pre-graft control, whilst the remainder was placed immediately into ice-cold media containing Liebowitz L-15 with glutamine, 10% fetal bovine serum, 1% penicillin/streptomycin and 1% non-essential amino acids (all Sigma, Poole, UK) for xenografting. Male CD1 nude mice (aged 4–6 weeks, n = 30; Charles River UK, Margate, England) were anesthetised by inhalation of isofluorane, and castrated. After a recovery period of 1–2 weeks, small pieces (1 mm^3^ approx.) of donor testis tissue were inserted subcutaneously under the dorsal skin using a 13G cancer implant needle (Popper and Sons, New York, US). Grafts (5–6 per mouse) were inserted on either side of the midline. Mice were housed individually and received analgesia (Carprofen; Pfizer, New York, USA) and antibiotics (Enrofloxacin; Bayer, Germany) in the drinking water for 5 days post-surgery. In general, 4–6 mice were xenografted with tissue from each fetus and half of these mice were then allocated to vehicle+hCG treatment and half to DES+hCG treatment.

### Treatments of Host Mice

Host mice receiving xenografts from human fetal testis tissue were injected subcutaneously every 72 h with 20 IU hCG (Pregnyl, Organon Laboratories, Cambridge, UK) in 0.9% (w/v) saline containing 1% (v/v) fetal bovine serum, which has been shown to be required to maintain testosterone production by the xenografts and reflects the high levels of hCG in human pregnancy [18]. Host mice were treated by subcutaneous injection with either vehicle (corn oil) or 100 µg/kg DES (Sigma, Poole, Dorset) diluted in corn oil. In xenografted host mice, all treatments commenced 7 days after grafting and continued 3 times a week until the end of the grafting period (∼5 weeks). Ungrafted castrate mice (n = 6) were also treated with either DES or vehicle as above in order to provide baselines for seminal vesicles and testosterone and to ensure that any effects of the treatment were specific to the human fetal testis xenografts. The dose and treatment regimen for DES was based on studies by ourselves (see below and [8]) and others [7], which showed major suppression of intratesticular testosterone in fetal rats and mice using this regimen.

### Retrieval of Xenografts

Xenografts of human fetal testis were retrieved after 6 weeks. Host mice were killed by cervical dislocation and blood obtained by cardiac puncture for measurement of testosterone. Seminal vesicles were removed and weighed and xenografts were retrieved, weighed and fixed for 2 h in Bouin’s fixative, before being transferred to 70% (v/v) ethanol and then processed into paraffin blocks.

### Fetal Rat Studies

Wistar rats were maintained according to UK Home Office guidelines and were fed a soy-free breeding diet (RM3(E) soya free; SDS, Dundee, Scotland). Housing conditions were carefully controlled (lights on at 0700, off at 1900 h, temperature 19–21°C, GOLD shavings and LITASPEN standard bedding (SPPS, Argenteuil, France)). Time-mated female rats were injected subcutaneously with DES (Sigma-Aldrich) at a dose of 100 µg/kg in 1 ml/kg corn oil on e13.5, e15.5, e17.5, e19.5 and e20.5. Rat dams were killed on e21.5 by inhalation of CO_2_ followed by cervical dislocation. Fetuses were removed, decapitated and placed in ice cold phosphate buffered saline (PBS; Sigma-Aldrich). Testes were microdissected, and fixed in Bouin’s fixative for 1 h at room temperature or snap frozen and stored at −70°C for gene expression analysis or determination of intratesticular testosterone (ITT). For the latter, the testis was homogenized and its total testosterone content determined after extraction and measurement as described below. Bouin’s-fixed tissues were processed and embedded in paraffin wax, and 5 µm sections were cut and used for immunohistochemistry.

### Testosterone Assay

Xenograft host serum testosterone levels and rat fetal intra-testicular testosterone levels were measured at termination by competitive radioimmunoassay using an in-house radioimmunoassay method described previously [21]. The method used radiolabelled testosterone (I^125^, MP Biomedicals, UK) and a rabbit primary antibody (1∶600,000; AMS Biotech, Abingdon, UK). Residual I^125^ was measured with a gamma counter (WIZARD 1470, Perkin Elmer, Turku, Finland) and testosterone levels were expressed as ng/ml (human xenografts) or pg/testis (rat fetal testis). All samples were analysed in a single assay, the detection limit for which was 45 pg/ml and the intra-assay CV was 8%.

### Immunohistochemistry

Immunohistochemistry was performed on human fetal testis tissue (ungrafted, n = 5; vehicle-exposed grafts, n = 3; DES-exposed grafts, n = 3), human adult endometrium obtained at endometrial biopsy (n = 1) and rat fetal testis (n = 3) as previously described [22]. Sections (5 µm) were subjected to heat induced antigen retrieval in 0.01 M citrate buffer (pH 6), and endogenous peroxidase blocked with 3% (v/v) H_2_O_2_ in methanol for 30 min. Endogenous biotin was blocked using an avidin/biotin blocking kit (Vector Laboratories Inc., Peterborough, UK), according to manufacturer’s instructions. Sections were incubated in normal rabbit serum diluted 1∶5 with TBS containing 5% (w/v) BSA for 30 min. Sections were incubated overnight with primary antibody (ESR1, mouse anti-human 1∶50; Vector Laboratories, Peterborough, UK) diluted in serum at 4°C in a humidified chamber and then incubated for 30 min with a biotinylated secondary antibody (rabbit anti-mouse; DAKO, Ely, Cambridgeshire, UK) at 1∶500, diluted in normal serum/TBS/BSA, followed by 30 min incubation with Streptavidin-HRP (DAKO) at 1∶1000, diluted in TBS. Visualization was performed using 3,3-diaminobenzidine tetrahydrochloride (DAB, DAKO) and sections were counterstained with hematoxylin before mounting in Pertex mounting medium (CellPath plc, Hemel Hempstead, UK). Negative controls were sections in which the primary antibody was replaced with the appropriate normal serum/TBS/BSA. Images were captured as previously described [22].

### Gene Expression Analysis

For quantitative analysis of gene expression by RT-PCR, total RNA was extracted from pre-graft control human fetal testis samples (n = 3) and adult human endometrium obtained at endometrial biopsy (n = 1) using the RNeasy Micro Kit with on-column DNase digestion (Qiagen, UK). Random hexamer primed cDNA was prepared using the Applied Biosystems Taqman™ RT kit (Applied Biosystems, CA). Quantitative real time PCR (qRT-PCR) was performed on the ABI Prism Sequence Detection System (Applied Biosystems). Expression of human *ESR1* was determined using the Roche Universal Probe Library (human *ESR1* forward primer 5′-AACCAGTGCACCATTGATAAAA-3′, reverse primer 5′-TCCTCTTCGGTCTTTTCGTATC-3′, probe number 69 Cat no. 04688686001; Roche Applied Sciences, Burgess Hill, UK). The expression level of human *ESR1* was corrected using 18S ribosomal RNA as internal control (Applied Biosystems Cat no. 4308329). All samples were performed in triplicate for 3 separate fetuses (14–19 weeks old) and a relative comparison was made to human adult endometrium for *ESR1*.

### Statistics

Statistical analysis was performed using Graphpad Prism 5 software (La Jolla, California USA). Results were analysed using paired t-test with Bonferroni post-hoc test. For human fetal testis xenografts data were also analysed by two-way ANOVA to take account of the variation between replicate grafts (ie grafts from the same fetus but into separate hosts that then receive comparable treatment) and between individual fetuses. Data for fetal ITT and mRNA levels were log transformed prior to analysis to normalise variances.

## Results

### Xenograft Retrieval from Vehicle- and DES-exposed Mice

**Figure 1:**
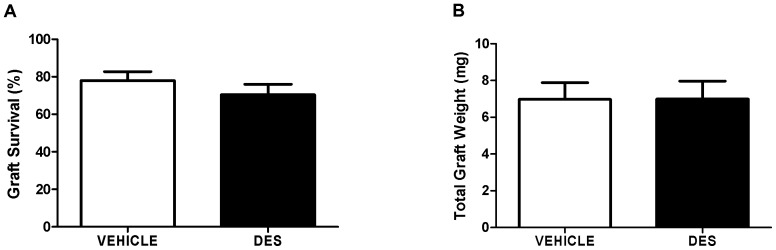
Graft survival and total graft weight of human fetal testis xenografts recovered from host mice exposed to either vehicle or DES at six weeks after xenografting. A) Graft survival (%). B) Total graft weight (mg). Data analysed by unpaired t-test; Mean ± SEM for n = 15, p>0.05.

At the end of the xenografting period, grafts were easily identified and retrieved from the subcutaneous tissue. The majority of the 182 grafts were retrieved at the end of the grafting period with no difference between vehicle or DES-exposed groups (78 v 70% retrieval, p>0.05; Fig. 1A). Total recovered graft weight per mouse was almost identical for vehicle- and DES-exposed groups (6.98 v 6.99 mg; Fig. 1B).

### Effect of Exposure to DES on Testosterone Production by Xenografts

**Figure 2:**
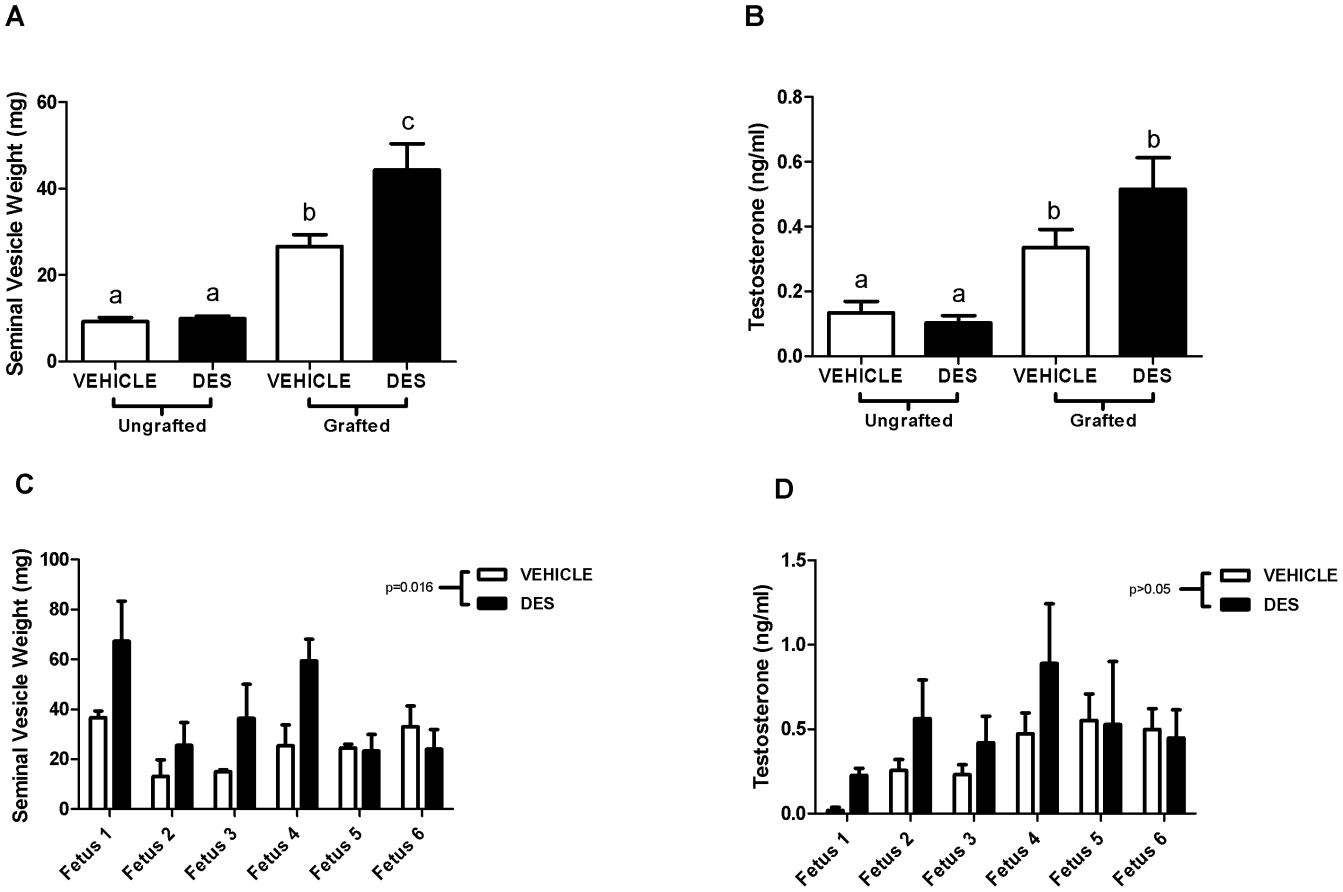
Effect of exposure to DES on testosterone production by human fetal testis xenografts. Seminal vesicle weight A) and serum testosterone B) in ungrafted (n = 6) and xenografted (n = 15) mice. Mean ± SEM. Bars with letters in common are not significantly different. Data analysed by unpaired t-test. Base-line variation in seminal vesicle weight C) and serum testosterone D) for host mice xenografted with individual fetuses (n = 6). Mean ± SEM. Data analysed by two-way ANOVA.

To determine the effect of exposure to DES on testosterone production from the xenografts, serum testosterone and seminal vesicle weight were measured in the host animals. Unexpectedly, seminal vesicle weight was significantly increased in the DES-exposed hosts compared to vehicle (26.6 v 44.3 mg, p = 0.002; Fig. 2A). Serum testosterone levels at termination were not significantly different in the DES-exposed hosts compared to vehicle (0.33 v 0.51 ng/ml, p>0.05; Fig. 2B). There was variation between individual fetuses for baseline seminal vesicle weight (Fig. 2C) and serum testosterone (Fig. 2D) and therefore two-way ANOVA was performed to take into account this variation. In order to ensure that these results were due to effects on the xenografts and not the host animal, we measured seminal vesicle weight and serum testosterone in ungrafted mice. There was no effect of DES on seminal vesicle weight or serum testosterone in ungrafted control mice (Fig. 2A,B)).

**Figure 3:**
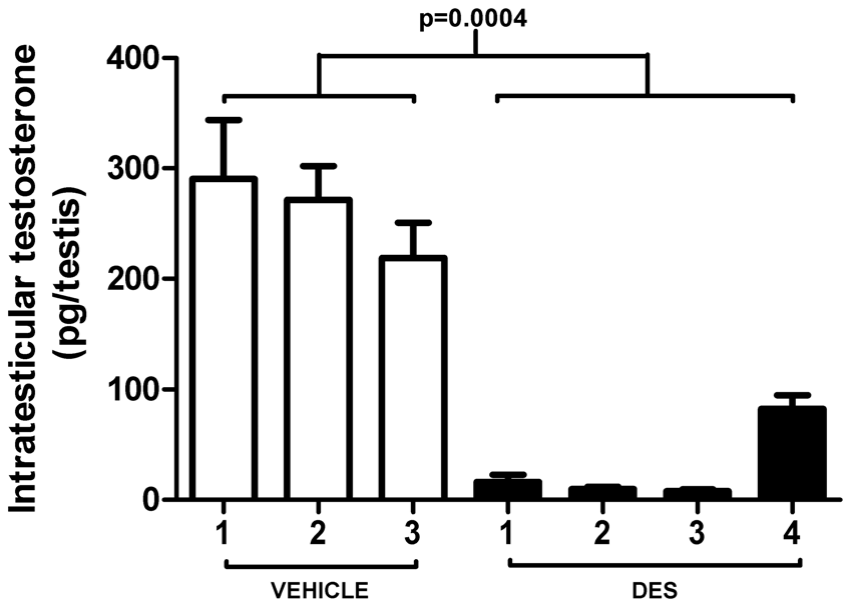
Effect of exposure to DES from e13.5–e20.5 on intratesticular testosterone (ITT) at e21.5 in fetal rat testes. Data is presented as litter means (unpaired t-test, p = 0.004).

To compare the results obtained for the human fetal testis with those in the rat, we exposed pregnant rats to DES (n = 4) or vehicle (n = 3) from e13.5– e20.5 and intratesticular testosterone was determined at e21.5; because of the small blood volume of e21.5 fetuses it was not feasible to measure blood levels of testosterone. There was a significant overall decrease (89%) in intratesticular testosterone in DES-exposed fetal rat testes compared to vehicle-exposed controls (260.2 v 29.1 pg/testis, p<0.001; Fig. 3). This finding was consistent between litters with a significant reduction in ITT in all DES-exposed litters compared with controls.

### ESR1 Expression in the Fetal Testis of Human and Rat

**Figure 4:**
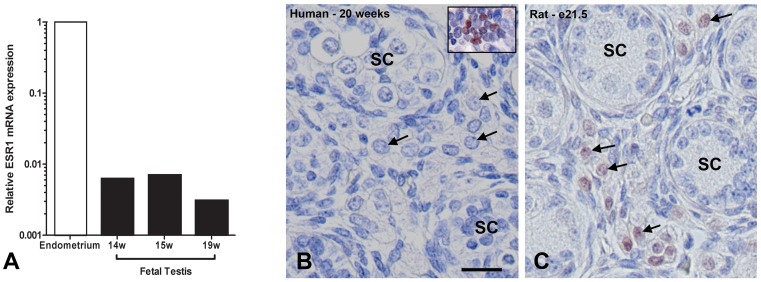
ESR1 expression in human fetal testis and endometrium. A) Relative *ESR1* mRNA expression in adult human endometrium compared with human fetal testis (n = 3). w = weeks. Note: data presented on a log-scale. ESR1 protein expression in B) a 20 week gestation ungrafted human fetal testis and human endometrium (positive control; inset.) and C) e21.5 rat fetal testis. SC = seminiferous cords, arrows indicate Leydig cells. Original magnification 20×. Scale bar = 50 µm.

The effects of DES in rodents have been shown to be mediated through *ESR1* signalling [9]. We compared ESR1 expression in control fetal human testis (n = 3) with that of rat. *ESR1* mRNA was barely detectable in the human fetal testis (Fig. 4A). Human fetal testis exhibited 175-fold lower mRNA expression compared with human adult endometrium. Immunoexpression of ESR1 protein was not detected in the Leydig cells of the human fetal testis (Fig. 4B). In addition no expression was identified in either vehicle-exposed or DES-exposed xenografts (not shown). In contrast, in rats, there was clear immunoexpression in fetal rat Leydig cells (Fig. 4C). As a positive control ESR1 protein expression was detected in the human endometrium demonstrating the specificity of this antibody in human tissue (Fig. 4B, inset).

## Discussion

The original ‘estrogen hypothesis’ [6] proposed that the increase in male reproductive abnormalities (testis cancer, cryptorchidism, hypospadias, low sperm counts) over recent decades might have occurred because of increased exposure to estrogens from various potential sources. Subsequent studies in rats and mice that involved manipulation of fetal estrogen exposure confirmed that supranormal estrogen exposure could act on steroidogenic pathways that can underlie male reproductive disorders [2], [7], [8]. However, direct evidence of an effect of DES on the human fetal testis has so far been lacking. The present studies aimed to determine the effect of exposure to DES on testosterone production by the human fetal testis using a xenograft approach, shown to recapitulate normal human fetal testis development and to be a useful model in which to determine the effects of exposure to proposed ‘endocrine disruptors’ [18], [19].

The present studies have demonstrated that exposure to DES, given at a dose in the equivalent range to previous clinical use (100 µg/kg three times a week), does not lead to any detectable reduction in testosterone production/action by second trimester human fetal testis xenografts. In fact we found a significant increase in seminal vesicle weight in xenografted host mice exposed to DES, indicating an increase in overall testosterone production from the xenografts during the grafting period, although there was no significant difference in single measurements of serum testosterone concentration at the time of graft recovery. The increase in seminal vesicle weight is unexplained and requires further study. In non-castrate adult mice exposed to DES, seminal vesicle weights are reported to decrease [23]. We have ruled out the possibility that a direct action of DES on the mouse seminal vesicles is causing the increase in seminal vesicle weight by exposing ungrafted castrate mice to DES and found no difference, therefore the increase in seminal vesicle weight in xenografted mice must involve the human fetal testis. Our results do not rule out the possibility that DES can affect testosterone production via indirect mechanisms that involve effects on the placenta or the fetal brain, however we consider it unlikely that the increased testosterone production is being stimulated centrally via the hypothalamo-pituitary-gonadal axis, given that high gonadotrophin levels induced by castration of the host animal have been shown to result in low (baseline) levels of testosterone production from the xenografts in the absence of exogenous hCG [19].

DES has been shown to exert its major adverse effects mainly through classical estrogen receptor signalling, in particular via ESR1 [24], [25]. In a mouse fetal testis organ culture system the reduction in testosterone production that occurred following DES exposure in wild-type testes did not occur in ESR1 deficient mice [24]. Given that the effects in mice are mediated by ESR1 we examined the expression of ESR1 in the human fetal testis and did not detect expression of this protein in fetal Leydig cells, consistent with previous findings [16], [17]. However, we confirmed that ESR1 expression was present in fetal rat Leydig cells; this difference offers a straightforward explanation for the differential sensitivity of fetal Leydig cells in terms of testosterone production following DES-exposure in rodents versus the human. It also indicates that any stimulatory effects on testosterone production in human fetal testes are likely to be mediated via an alternative estrogen signalling pathway. This is supported by evidence for actions of DES involving alternative pathways, such as the estrogen-related receptors [26] and/or the orphan receptor Nr0b2 [27].

We confirmed that exposure of the fetal rat testis to the same DES treatment regimen resulted in ∼90% reduction in fetal intra-testicular testosterone [7], [8]. As a reduction in testosterone production by the fetal testis is widely viewed as being the most likely mechanism for the origin of TDS disorders, the present findings suggest that exposure of the human fetal testis to estrogenic environmental chemicals, or indeed to any supranormal estrogen exposure, is unlikely to induce such effects, at least where this is mediated via ESR1.

Our findings are consistent with a recent study that used *in vitro* culture of human 1^st^ trimester fetal testes, which also showed that testosterone production was not reduced by exposure to DES [28]. The limitations of the *in vitro* approach, such as the short duration of exposure and decline in tissue integrity, are addressed by the current approach which permits long term treatments and maintenance of tissue integrity in addition to exposure via the circulation of the host animal [19].

From the 1940’s until the early 1970’s DES (∼60–70 µg/kg daily from gestational weeks 8–14, increasing by 5 mg/week thereafter), was prescribed to pregnant mothers for its proposed role in reducing spontaneous miscarriage and pre-term labour [10], [29]. It was subsequently shown that DES exposure *in utero* had a causal role in the development of vaginal malignancy [30]. It has also been reported that DES-exposure is associated with structural abnormalities of the male reproductive tract including epididymal cysts, microphallus and testicular hypoplasia (Reviewed in [29]). However there is conflicting evidence regarding DES and the disorders that comprise TDS. Several studies report an increased risk of testicular cancer following prenatal exposure to exogenous estrogen [31], [32], [33], [34], [35]. Of the two studies which specifically investigated DES effects, one found no increased risk [36]. All the other studies reported increased risks that were not statistically significant [31], [32], [33], [34], [35]. To date there remains no conclusive evidence of an increased risk of testicular cancer following prenatal DES exposure [6], [14], [29]. Hypospadias has been reported to be more prevalent in DES exposed males [37], however these data related to urethral abnormalities rather that hypospadias per se [38]. A recent meta-analysis reported a small increased risk of hypospadias in DES/estrogen exposed males [14]. However, any such effect may perhaps result from direct effects on the developing penis [39] [40], rather than as the consequence of reduced testosterone production by the fetal human testis. In addition the available epidemiological evidence suggests that human exposure *in utero* to estrogens other than DES, such as environmental estrogens and those in the oral contraceptive pill do not result in increased risk of TDS disorders in male offspring [14], [41].

A more recent study has shown an increased risk of cryptorchidism in males exposed *in utero* to DES, although this was associated with initial exposure before the 11^th^ week of gestation with no significant effect in those initially exposed after 11 weeks [15]. Rat studies have demonstrated that testosterone production/action within a discrete masculinization programming window (MPW), is responsible for programming subsequent normal male reproductive development, and TDS disorders may only result if there is a reduction in androgen production/action in this period [42]; the present studies investigated effects of DES exposure on the human testis from 15 weeks of gestation, which is after the postulated MPW [42]. It is therefore possible that this could explain the lack of effect of DES on testosterone production. However, the effects of DES on testosterone production by the fetal testis in rats occur beyond the MPW, as shown in the present study (e21.5). In addition, DES exposure of first trimester human fetal testes *in vitro* had no effect on testosterone production [28]. Taken together, it seems unlikely that our present negative findings regarding DES effects are simply due to the gestational age of the fetus. An alternative mechanism for the reported increased incidence of cryptorchidism could be via effects on Insl3, a key factor in testicular descent in rodents [43], [44]. In humans, Insl3 is believed to be important during early testicular descent between 8–15 weeks gestation [45]. Further studies to investigate the effects of DES on Insl3 in first trimester human fetal testis would address this.

### Conclusion

In rodents, exposure to DES *in utero* results in disorders of the reproductive tract mediated by a reduction in testosterone production. It has been postulated that similar effects occur in humans. Using a xenograft approach, we have demonstrated that exposure to DES does not result in a reduction in production of testosterone by the human fetal testis. This lack of effect on testosterone may be due to the lack of ESR1 in human fetal Leydig cells, in contrast to rodents. The findings highlight important species differences between humans and rodents, with implications for assessing the risk to human health that fetal exposure to environmental estrogens might pose.
